# Translation of a Host Blood RNA Signature Distinguishing Bacterial From Viral Infection Into a Platform Suitable for Development as a Point-of-Care Test

**DOI:** 10.1001/jamapediatrics.2020.5227

**Published:** 2021-01-04

**Authors:** Ivana Pennisi, Jesus Rodriguez-Manzano, Ahmad Moniri, Myrsini Kaforou, Jethro A. Herberg, Michael Levin, Pantelis Georgiou

**Affiliations:** 1 Department of Infectious Disease, Faculty of Medicine, Imperial College London, London, United Kingdom; 2 Centre for Bio-Inspired Technology, Department of Electrical and Electronic Engineering, Imperial College London, London, United Kingdom

## Abstract

This study assesses a 2-gene RNA signature that can be translated into a rapid (<25 minutes) and portable laboratory-on-a-chip platform suitable for development as a point-of-care test.

Discrimination of viral from bacterial infections remains a challenge, resulting in unnecessary investigation, admission, and antibiotic treatment of many patients with fever. Studies in *JAMA* previously reported that children with bacterial and viral infection can be distinguished by their blood host RNA signature.^1,2^ Here, we demonstrate that a 2-gene RNA signature can be translated into a rapid (<25 minutes) and portable laboratory-on-a-chip platform suitable for development as a point-of-care test.

## Methods

Herberg and colleagues^1,3^ reported a 2-transcript signature (*IFI44L* and *FAM89A*) discovered using gene expression microarrays in a set of 455 children with fever and bacterial and viral infections (Inflammatory and Infectious Disease Study [IRIS] study). We randomly selected 24 RNA samples from patients in the IRIS study^1^ with confirmed bacterial (n = 12) and viral (n = 12) infections, matched for severity, collected between September 2009 and May 2017, and extracted using the PAXgene Blood RNA Kit (PreAnalytiX GmbH). We identified transcripts suitable for translation to our laboratory-on-chip platform, which uses reverse transcription loop-mediated isothermal amplification (RT-LAMP), by assessing counts of *IFI44L* and *FAM89A* in a publicly available blood RNA sequencing data set comprising 255 children with bacterial and viral infections (GSE69529).^4^ The average gene counts for *IFI44L* were sufficient (2281.9) but low for *FAM89A* (20.2), potentially compromising transferability across platforms. Therefore, using the list of previously identified 38 highly correlated transcripts,^1^ we replaced *FAM89A* with *EMR1-ADGRE1* (RefSeq ID: NM_001974.5), which had sufficient average gene counts (511.3),^4^ and in combination with *IFI44L* (RefSeq ID: NM_006820) had a similar performance to the original 2 transcript signature (area under the curve [AUC] in the training, test, and validation data sets: 93.4%, 97.4%, and 97.2%, respectively).

**Figure 1.**
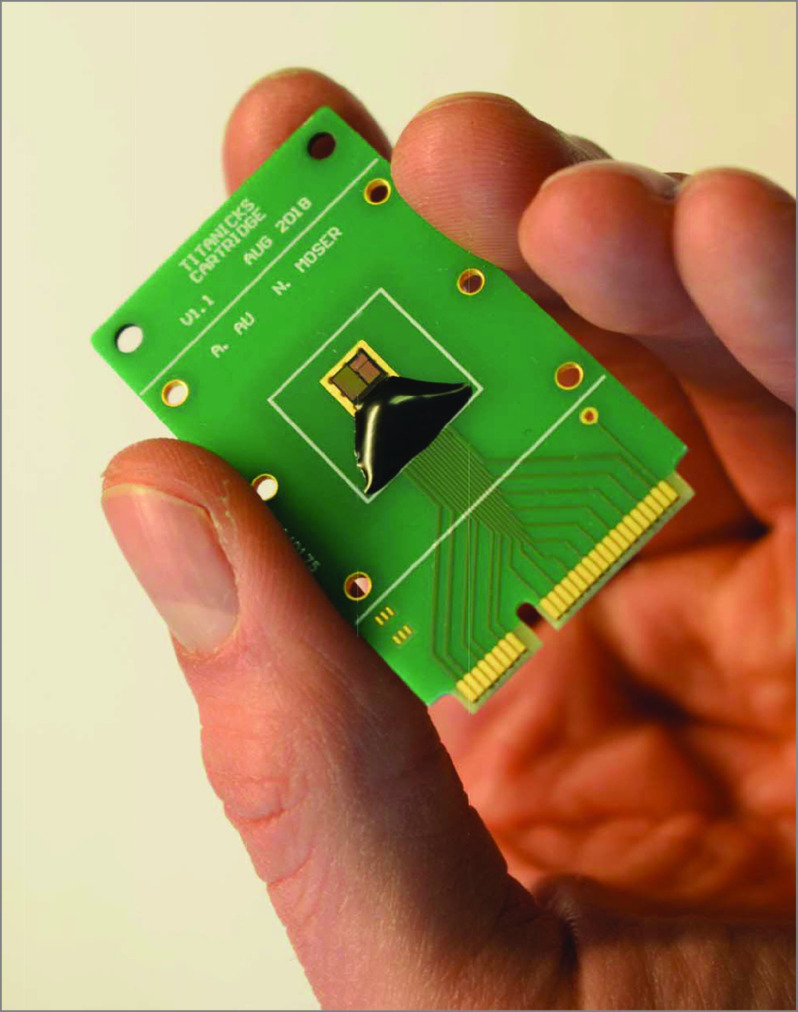
A Handheld Point-of-Care Test Disposable diagnostic cartridge containing a complementary metal-oxide semiconductor ion-sensitive field-effect transistor array for on-chip real-time nucleic acid amplification.

Our laboratory-on-chip platform combines novel pH-sensing complementary metal-oxide semiconductor technology^5^ with RT-LAMP, which we term *electronic RT-LAMP* (RT-eLAMP) (Figure 1). RT-eLAMP uses thousands of microsensors (ion-sensitive field-effect transistors) to detect H^+^ ions released, resulting in a change in pH, during the nucleic acid amplification process following the same experimental conditions as reported in our previous article.^6^

We compared the gene expression values (normalized log_2_ fluorescence for microarrays^1^) or time-to-positive values from signals of all the microsensors for RT-eLAMP using support vector classifiers, with 10-fold cross-validation, and each patient was assigned a score reflecting risk of bacterial or viral disease (package: scikit-learn in Python). We evaluated the predictive accuracy of the score in patients with microbiologically confirmed diagnoses using receiver operating characteristic curves, AUC, and 95% CIs under the binomial distribution. Analysis began April 2020.

## Results

**Figure 2.**
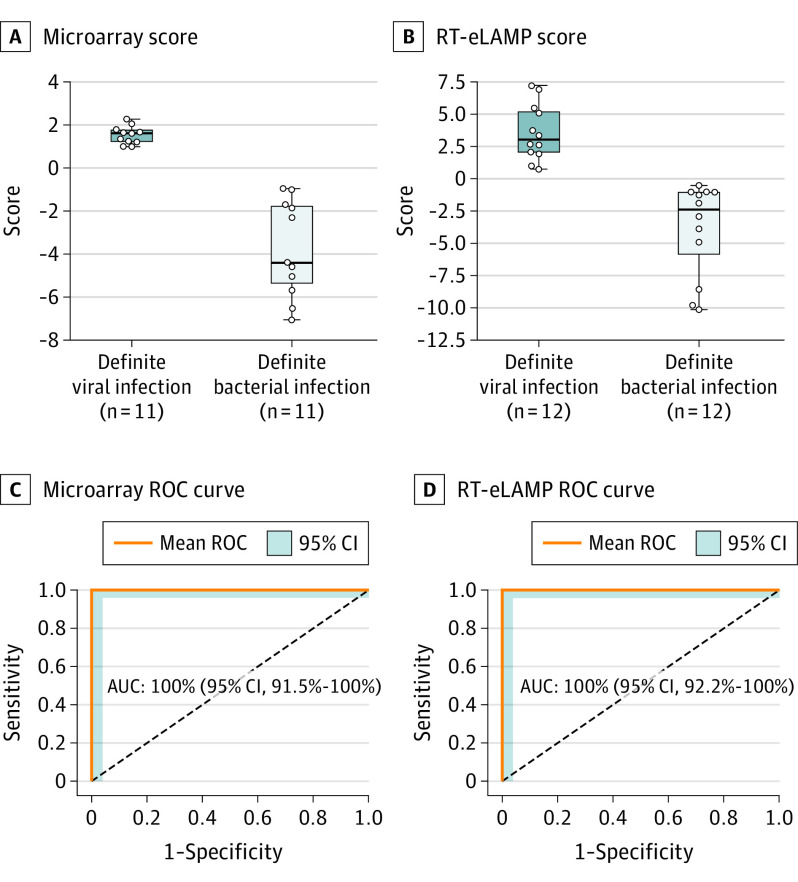
Performance of the 2 Diagnostic Genes in Microarray and Electronic Reverse Transcription Loop-Mediated Isothermal Amplification (RT-eLAMP) Predicitive score, receiver operating characteristic (ROC) curves, and expression values based on the 2-transcript signature for distinguishing definite viral (dark blue) and definite bacterial (light blue) samples. Boxes indicate the interquartile ranges and the median (middle line). Whiskers indicate 1.5 times the interquartile range. A and C, Performance using expression values obtained from the microarray platform. B and D, Performance using our RT-eLAMP platform. AUC indicates area under the curve.

Using microarray data,^1^ the 2-transcript signature applied to children with bacterial and viral infections had a sensitivity and specificity of 100% (95% CI, 95.7%-100%) and 100% (95% CI, 95.7%-100%), respectively, with AUC of 100% (95% CI, 91.5%-100%) (Figure 2A and B). Translating the 2-gene signature to RT-eLAMP showed similar results: sensitivity and specificity were 100% (95% CI, 96.0%-100%) and 100% (95% CI, 96.0%-100%), respectively, with AUC of 100% (95% CI, 92.2%-100%) (Figure 2C and D). The calculated limit of detection for the 2 assays were 10 RNA copies per reaction in 10-μL reaction volume and time to positive under 25 minutes, much faster than microarray.

## Discussion

Previously reported RNA signatures that differentiate bacterial from viral infection were discovered using cumbersome transcriptomic technologies. We have moved the promising transcript signatures closer to clinical application by establishing that the 2-transcript signature can be detected using a semiconductor-based sensing platform combined with isothermal amplification chemistries. The absence of fluorescent labels and the economy of scale of the microchip industry makes the technology potentially suitable for implementation at low cost (<£1 [US $1.33] per chip). While our study includes modest patient numbers, it provides a proof of concept that host RNA signatures can be detected rapidly and cost-effectively in a format suitable for development as a point-of-care diagnostic test that might be applied to a range of clinical diagnoses. This study has a number of limitations including a relatively small sample size, a focus on confirmed infections, and that the platform currently lacks integrated sample preparation and has a limited numbers of wells per microchip.
